# Can snow depth be used to predict the distribution of the high Arctic aphid *Acyrthosiphon svalbardicum *(Hemiptera: Aphididae) on Spitsbergen?

**DOI:** 10.1186/1472-6785-11-25

**Published:** 2011-10-13

**Authors:** María L Ávila-Jiménez, Stephen J Coulson

**Affiliations:** 1 Dept. Arctic Biology, University Centre in Svalbard, P.O. Box 156, 9171, Longyearbyen, Norway; 2 Ecological and Environmental Change Research Group (EECRG), Department of Biology, University of Bergen, P.O. Box 7800, N-5200 Bergen, Norway

## Abstract

**Background:**

The Svalbard endemic aphid *Acyrthosiphon svalbardicum *(Heikinheimo, 1968) is host specific to *Dryas octopetala *L. ssp *octopetala *(Rosaceae). It has been hypothesized that the aphid is present on those areas with a thin winter snow cover and which therefore clear of snow earlier in the season. This early snow clearance results in a longer growing period and allows the aphid to experience at least the minimum number of degree days required to complete its life cycle. However, this hypothesis lacked a detailed field validation. We aimed to test the relationship between the aphid distribution and time of snow clearance at landscape scale, mapping snow depth at peak of snow accumulation for the two succeeding years 2009 and 2010 and examining site occupancy and plant phenology the following summers. Additionally, the distribution range mapped by Strathdee & Bale (1995) was revisited to address possible changes in range along the coast of the fjord.

**Results:**

A linear relation between snow depth and timing of snow melt was found but with strong inter-annual and landscape variation. Both snow depth and plant phenology were found to affect patch occupancy. In August, the aphid, at the three life stages scored (viviparae, oviparae/males and eggs), was present most frequently in those *D. octopetala *patches with the most advanced plant phenology and which showed shallower snow depths in spring. However, many patches predicted to contain aphids were empty. The aphid distribution range has expanded 4.7 km towards the fjord mouth from 1995.

**Conclusions:**

Snow depth alone, and hence date of snow clearance, cannot precisely define species distribution at landscape scale, as this cannot explain why are they unoccupied patches under shallow snow depths with advanced plant phenology. We nonetheless present a model Arctic system that could form the basis for long term monitoring for climate- driven species shifts.

## Background

The climate envelope paradigm (the range of climatic requirements or tolerances) [1] is often criticised as a simplistic approach in modelling species distributions as it systematically excludes the influence of biotic factors on species distributions [2,3]. The inherent difficulty in precisely defining the factors determining the distribution range of a species can be particularly misleading when predicting shifts in the species range as a response to climate change [2]. Nonetheless, the fine resolution of the factors affecting successful reproduction and dispersal abilities is essential for predicting the effects of environmental change on species distribution [3]. For Arctic species strongly dependant on the number of degree days to successfully complete their life cycle, factors such as length of summer, temperature, timing of snow melt, snow thickness and overwintering conditions [4] are all likely to influence species presence or absence. Surface ice layer thickness has also been shown to have a deleterious effect on soil invertebrate populations [5].

Depth of snow, and hence date of snow clearance and length of the summer period, is intuitively important in determining species occurrence. For many species, especially holocyclic species, the date of snow clearance may be critical. However, few studies have investigated this point with the exception of the research by Strathdee & Bale [6] on the Svalbard endemic aphid *Acyrthosiphon svalbardicum *(Heikinheimo, 1968), and studies regarding snow bed plant communities [7-10]. Climate predictions point to an increase in winter snowfall combined with increased winter snow evaporation, leading to as yet uncertain changes in the timing of the snow melt [11]. In the case of *A. svalbardicum*, knowledge of the environmental factors controlling its biology and distribution is key to better understand the abilities of the species to track its ecological niche in changing times [12]. Predicting species distribution response to environmental changes can stand as a major challenge if complex relations are to be modelled. For instance, plant phenology can also be considered among the same environmental factors that affect aphid distribution as temperature, snow thickness [7,13] summer length, or timing of snow melt [14]. Hence identifying the direct effect of a single isolated environmental variable can be a challenging task. Specialist phytophagous species often show a strong phenological synchronization with host plant [15-17]. Furthermore, host plant distribution, growth and phenology can be directly influenced by temperature. For example low summer temperature limit seed setting in the high Arctic [18], vegetative growth and germination are positively affected by warming, and peak flowering can be brought forward by increasing temperatures [19].

*Acyrthosiphon svalbardicum *is host specific to *Dryas octopetala *L. ssp *octopetala *(Rosaceae). However, the distribution of the aphid is more restricted than the distribution of the host plant, that is, not all patches of *D. octopetala *harbour aphids. Notable studies regarding the environmental factors effecting aphid distribution in Kongsfjord (Svalbard archipelago) were carried out during the early 1990's [6,20], concluding that the aphid was restricted to those areas which clear up from snow earlier in the season and where the aphid will experience the minimum number of degree days required to complete its annual life cycle [6]. This simplistic hypothesis, the direct relation between snow depth and the aphid distribution, was rapidly entrenched in polar science, becoming a modern axiom in Arctic ecology, even though the field study was constrained to six *D. octopetala *patches in a 2.7 by 3.5 m south facing plot in a single location within the settlement of Ny-Ålesund [6]. Verification regarding the representativeness of that patch on the species range is lacking, even though the likelihood of misestimating the effect of unusual events is recognized to increase with the reduction on the scale of the study [21]. In fact, later studies in the southern location of Endalen (close to Isfjord) have showed the aphid to be more abundant than expected under deep snow profiles [22]. Consequently, whether snow depth, and hence timing of snow clearance, has a role in determining the local limit of the distribution of the aphid yet remains unclear.

*Acyrthosiphon svalbardicum *is locally abundant on coastal ridges along the south coast of Kongsfjord, where its occurrence has been described as decreasing towards the entrance of the fjord and with distance from the shore [6], even though the host plant is ubiquitous in the area. This species is thought to have low dispersal abilities since the production of alate forms appears to be rare [20]. This has led to the hypothesis that this species may not be able to rapidly track suitable environmental conditions but that it is restrained to those few consistently microclimatically favourable sites [23]. *Acyrthosiphon svalbardicum *is a holocyclic species that overwinters as an egg, and the complex life cycle includes four morphs (fundatrix, vivipara, male and female). The fundatrix gives rise directly to both sexual morphs and a viviparous form. The viviparae produces an additional generation of sexual morphs which often fails to give rise successful offspring, but which should it succeed, will increase the number of overwintering eggs by 11 fold [20]. The production of alate forms of the aphid has been rarely reported, and this event is thought to be related to unusually long or warm summers [6,24]. All the life stages of the aphid occur on *D. octopetala*, where it is more common on shoots with seed heads or flower buds than on vegetative shoots. The eggs are often found on the underside of the leaves [6].

Set against this background, we aimed to: 1) assess the relationship between snow depth and the distribution of *A. svalbardicum*; 2) survey for changes in the local range since 1995; and 3) combine field data on ice and snow depth, timing of snow melt, patch occupancy, aphid phenology and host plant phenology to determine possible relationships between these environmental variables and the distribution of the aphid at landscape scale. Moreover, we aimed to generate a baseline distribution and environmental map available for future studies both regarding snow depth and species distribution.

## Results

### Snow depth and the timing of snow clearance

**Figure 1:**
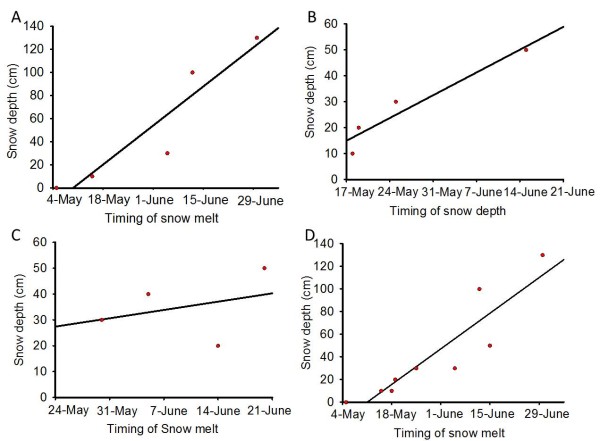
**Timing of snow melt in relation to snow depth**. **A: **south slope 2009; **B: **south slope 2010; **C: **north slope 2010; **D: **2009 and 2010 south slope data pooled together.

A clear increase in temperature fluctuations indicating timing of snow melt was recorded by all temperature loggers (see additional file 1). The temperature data revealed a linear relation between snow depth and timing of snow melt on south facing slopes (Figure 1), although the relation varied slightly between 2009 (R^2^= 0.87; p < 0.05) (Figure 1A) and 2010 (R^2^= 0.91; p < 0.05) (Figure 1B). There was no significant relationship for north facing slopes (R^2^= 0.11; p > 0.05) in 2010 (Figure 1C). Additionally, the relationship slightly weakens when multiyear data from south facing slopes is pooled together (R^2^= 0.82; p < 0.001) (Figure 1D). The earliest melting date recorded was 5^th ^May 2009 for areas with < 5 cm of snow in April, and the latest melting date recorded was 30^th ^June 2009 for areas with 130 cm of snow in April.

**Figure 2:**
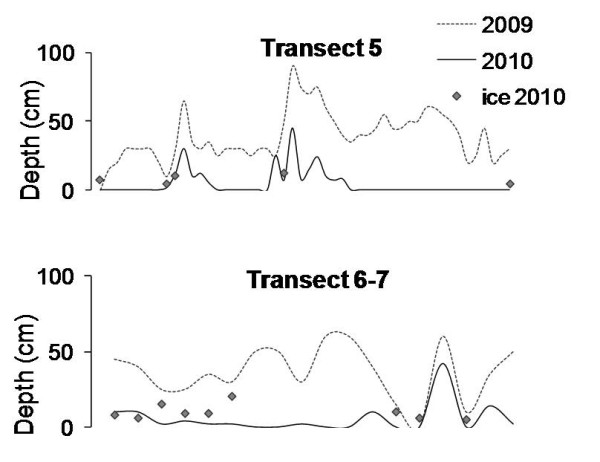
**Inter-annual variation in snow depth**. Snow depth measured along transects 5 and 6-7 in 2009 and 2010. Dotted line: snow depths in 2009; solid line: snow depths in 2010. Grey dots represent ice thickness measured in 2010.

Snow depth data showed a strong inter-annual within-site variation. Snow depth at some sites in 2009 was up to 67 cm thicker than in 2010 (Figure 2). The surface ice layer also showed a strong variation. In 2009, the surface ice layer measured on snow-free patches was up to 2 cm thick, but disappeared when snow depths reached 10 cm or deeper. However, in 2010 the surface ice layer was considerably thicker, varying from 4 cm in snow free areas to 27 cm under 50 cm of snow.

The minimum soil temperature recorded reached -17.6°C (recorded on the 16^th ^March 2010 by the logger placed in a south facing site under 30 cm of snow).

### Aphid phenology

A significant relation was found between aphid phenology and snow depth (H = 11.890, d.f. = 2, p < 0.05), but not between aphid phenology and host plant phenology (H = 5.492, d.f.= 2, p > 0.05). Although aphids were found mostly on those patches with the most advanced phenological stage, the eggs were found almost equally on plant stages 4 and 5 (Table 1). Eggs were present mostly on those areas showing shallower snow depths in winter, while viviparae and oviparae forms were found at a range of depths. Fertilized eggs (black and shiny) were observed on 10 occasions confirming successful reproduction. Viviparae forms were scored 11 times while oviparae and males were scored in 30 occasions.

**Table 1:** Patches occupied and aphid phenology in relation to plant phenology.

	Plant Phenology				
	**1**	**2**	**3**	**4**	**5**

**Total *D. octopetala *patches with the given phenology**	6	46	10	74	146
**Patches occupied**	0	4	0	19	59
**Patches free of aphids**	6	42	10	54	87
**% Patches occupied**	0	8	0	35	67
**% Oviparae scored**	0	0	0	16	83
**% Viviparae scored**	0	9	0	9	81
**% Eggs scored**	0	0	0	55	44

(0: No bud visible; 1: Stamens visible; 2: Petals senescing; 3: Seed head visible above petals; 4: Seed head untwisting; 5: Seed head twisting). The number of patches occupied is given as absolute figure in relation to the 282 patches surveyed, and as a percentage of the total number of patches with the given phenology that are occupied by the aphid. Additionally the percentage of each aphid life stage (from the scored oviparae/male, viviparae and eggs) scored at each plant phenological stage is presented.

### Host plant phenology

Host plant phenology was examined in August 2009 in 282 patches of *D. octopetala*, 52 patches still bore flowers, while a total of 230 patches showed seed heads in other developmental stages. In August 2009, 11 patches had no visible seed or flower bud. The most common phenological stage at that time of the year was stage 5 (seed head twisting) with 146 patches of *D. octopetala *at this stage (Table 1). Host plant phenology showed no relationship with orientation of the patches (north, south slope or top of the ridge) (H = 44.23; df = 2; p > 0.1). However, a significant relationship between snow depth and plant phenology was found (H = 359.8; df = 1; p < 0.001), and the earlier phenological stages were found on areas with deeper snow profiles in winter.

### Patch occupancy and aphid distribution range

From 594 points analysed in 2009, 339 represented a patch of the *D. octopetala*, and 98 of those were occupied by the *A. svalbardicum*.

From the 24 *D. octopetala *patches re-visited in 2010, 2 patches occupied in 2009 were now empty and 2 of the empty ones were re-colonized in 2010. The occurrence of the aphid was confirmed at the end points surveyed by Strathdee *et al*. [6].

### Distribution of the aphid on the host with relation to snow depth and host phenology

**Figure 3:**
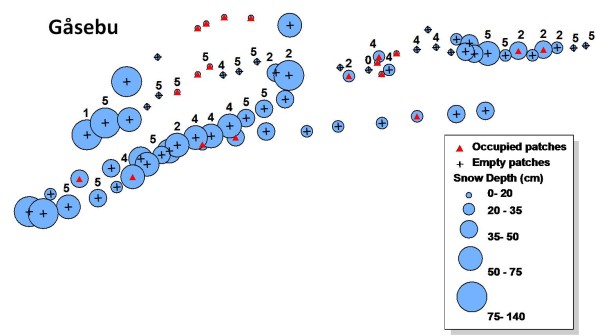
**Distribution map**. Distribution of *A. svalbardicum *along three transects on the Gåsebu ridge with snow depth and host plant phenology. Map shows the snow depths measured in April 2009 (blue circles), plant phenology recorded in August 2009 (0: No bud visible; 1: Stamens visible; 2: Petals senescing; 3: Seed head visible above petals; 4: Seed head untwisting; 5: Seed head twisting), and site occupancy recorded in August 2009 (Red triangles: occupied patches; Crosses: empty patches) in transects 6, 7 and 03.

**Figure 4:**
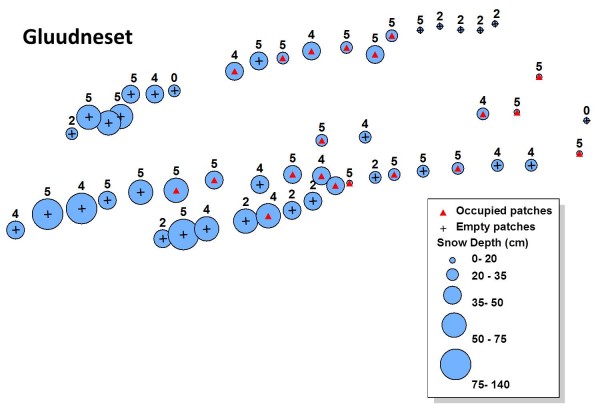
**Distribution map**. Distribution of *A. svalbardicum *along three transects on the Gluudneset ridge with snow depth and host plant phenology. Map shows the snow depths measured in April 2009 (blue circles), plant phenology recorded in August 2009 (0: No bud visible; 1: Stamens visible; 2: Petals senescing; 3: Seed head visible above petals; 4: Seed head untwisting; 5: Seed head twisting), and site occupancy recorded in August 2009 (Red triangles: occupied patches; Crosses: empty patches) in transects 9, 10 and 12.

**Figure 5:**
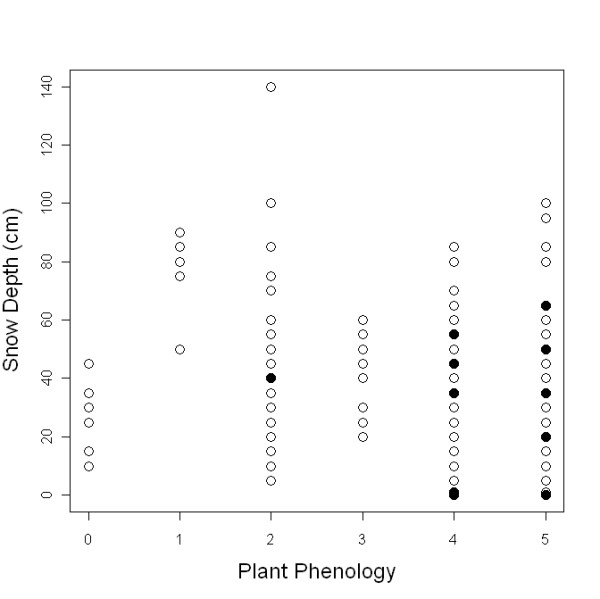
**Patch occupancy**. In the figure patch occupancy is represented in relation to snow depth and plant phenology. The graph represents the patches occupied by the aphid (filled circles) and empty patches (blank circles) in relation to snow depth (measured in April) and plant phenology (measured in August) (0: No bud visible; 1: Stamens visible; 2: Petals senescing; 3: Seed head visible above petals; 4: Seed head untwisting; 5: Seed head twisting).

**Figure 6:**
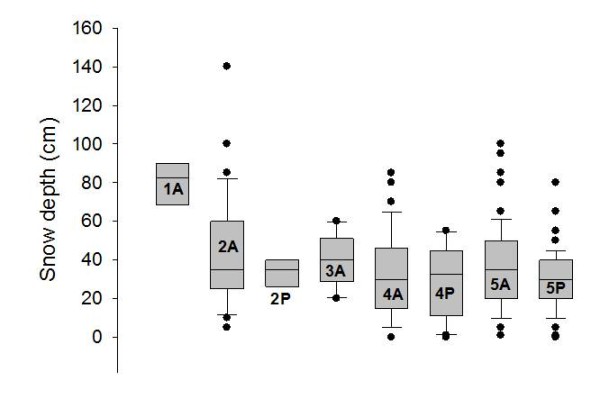
**Range of snow depth measured at each phenological stage on occupied and unoccupied patches**. The box plot shows snow depth measured in April for those patches where aphid presence (P) or absence (A) was recorded the following August, grouped by phenological stages (0: No bud visible; 1: Stamens visible; 2: Petals senescing; 3: Seed head visible above petals; 4: Seed head untwisting; 5: Seed head twisting), with standard deviation (solid bars) and outliers (dots).

Snow depth, site occupancy and plant phenology were mapped for both ridges (Figure 3, for transects 6 and 7 and Figure 4 for transects 9, 10 and 12. See additional file 2). Detailed snow depths and species distributions were combined to determine the maximum snow depth where the aphids occurred in 2009. The aphid was present at snow depths of up to 80 cm for plants in the most advanced phenology (5 -seed head twisted-) and 60 cm for less advanced host plant phenological stages. No aphids (or eggs) were found in *D. octopetala *patches on early phenological stages (white petals, stamens visible) (Figure 5) (Table 1). Overall, the aphid was restricted to those patches at phenological stages 2, 4 and 5. Just 8% of *D. octopetala *patches at stage 2 were occupied by the aphid but the percentage of occupied patches increased to 35% and 67% for stages 4 and 5 respectively (Table 1). A binomial linear model showed a significant relationship between snow depth and site occupancy (z = -2.89, p < 0.01) and host plant phenology and site occupancy (z = -5.61, p < 0.001). Significant differences were shown between the snow depth measured for occupied (P) and un-occupied (A) patches for each host plant phenological stage (Figure 6) (H = 24.9; df = 7; p < 0.001). A Dunn's *post- hoc *for pairwise differences revealed no significant differences in snow depth measured for occupied (P) and un-occupied (A) patches for each host plant phenological stage (Figure 6). Significant differences were found, however, between snow depths measured at patches in the earlier phenological stage (1A), and patches at the later phenological stages: 4A, 4P (occupied -P- and un-occupied -A- patches on stage 4) and 5P (occupied -P- patches on stage 5) (Q = 3.58, 3.85, and 4.1 respectively; p < 0.05) (Figure 6).

## Discussion

Species distribution modelling is a fast advancing field [25], with applications that reach from population monitoring [26], assessing climate driven species range shift [27,28], conservation biology [29], to testing biogeographical, ecological and evolutionary hypotheses [30]. It is often possible to predict a link between climate and species distributions [31], although the application of climate envelope paradigms in the modelling of species distributions have repeatedly raised the need to include a range of factors/tolerances beyond only climatic variables [2,3]. In the case of the Svalbard endemic aphid *A. svalbardicum*, a high Arctic species strongly dependant on number of degree days to successfully complete its life cycle, summer temperature and length of the growing season are thought to be limiting factors governing the distribution of the species [6,20]. Previous studies indicated snow depth as an accurate predictor of *A. svalbardicum *distribution [6], indicating that the aphid succeeds in reproduction, and thus is commonly present, in those patches with snow clearance early in the season and where the aphid would experience at least the minimum number of degree days required to complete its life cycle. In our study we compared multiple transects over a series of ridges and did observe at a larger scale the predictions made by Strathdee & Bale [6]. Eggs were found in those patches that showed shallower snow depths in winter, although the aphid was more widely distributed across areas that showed a range of snow depths. Our study also shows a statistically significant relationship between plant phenology and site occupancy that was previously overlooked, and also between plant phenology and snow depth leading to a much more complex picture. Due to strong correlation between all measured variables, neither this study, nor the previous, can infer causality between snow depth and the aphid distribution. Although the prediction by Strathdee & Bale [6] fits almost perfectly along a particular transect (transects 9, 10 and 12) (Figure 4), the relationship cannot be generalized for the species range across the coastal ridges in Kongsfjord. This was also the case for the distribution of the aphid in Endalen (close to Longyearbyen in Adventdalen) along a natural snow depth transect, where Dollery *et al*. [22] found a relationship between snow depth and the aphid distribution, demonstrating that shallow snow depths favour high aphid densities but while a thick winter snow pack does not preclude aphid presence. Our survey also reveals a number of cases where the species would be predicted to occur based on the Strathdee & Bale [6] hypothesis but where it does not actually occur (Figure 3, 4 and additional file 2). Our results show a greater number of suitable (shallow snow) patches unoccupied by the aphid than occupied. They also show site occupancy at a range of snow depths and a strong relationship between site occupancy and the phenology of the host plant.

Specialist phytophagous species sometimes rely on plant secondary metabolites as sign of stimuli during host selection, setting and parturition [32]. Synchronization with the host plant is often essential for neonate success after hatching [15,17]. Phenological synchronization between phytophagous species and their host plants is a common phenomenon [15,17,31], as in the case of the Arctic aphid *Sitobion calvulus *Ossiannilson, 1958, whose life cycle appears to be closely synchronized (genetically programmed) with its host plant *Salix polaris *[16]. The phenology of *D. octopetala *nonetheless can suffer shifts even within one season due to changes in snow accumulation [33]. In our study the aphid was found mostly in patches showing an advanced phenology, independently from the snow depth they experienced the previous winter. However, even a combination of snow depth (distribution predictor by Strathdee & Bale [6]) and plant phenology (additional species predictor described in the present study) cannot predict real site occupancy at a landscape scale (Figure 3, 4 and additional file 2). Strathdee & Bale [6] used a probabilistic approach, which although gave an insight on environmental factors affecting species distribution, cannot be used to model accurately the actual distribution range of the species. The significance of the relationship between aphid presence and plant phenology however should be interpreted with some caution due to the skew of the plant phenotype data resulting from low sample sizes from early phenological stages.

How environmental factors affect species biology, from physiology to biogeography, is also often simplified by a pragmatically reduced knowledge on functioning and dynamic of the physical environment. Even though within the same year and aspect (Figure 1A and 1B) there is evidence of a direct relationship between snow depth and timing of snow melt, this relationship weakens when the inter-annual data are considered and disappeared completely for north facing slopes. Snow depth cannot therefore be considered as a distribution predictor on north facing slopes because of the lack of any relationship between snow depth and the timing of snow melt. Variation in the timing of snow melt can be as blunt as the difference between the 100 cm of snow that melted 12^th ^of June 2009 and the 20 cm of snow that melted with just 2 days difference (14^th ^June) a year later; emphasizing the need for fully replicated multi-year studies and the difficulty involved in resolving cause and effect relationships.

Our data cannot yet explain why there are un-occupied patches even at shallow snow depths and possessing a suitable phenological stage. Even if aphid presence was overlooked in very low density patches that would also imply very low density patches are occurring at putative ideal patches. Inter-annual variations in snow depth, ice thickness, length of summer, plant phenology and predation pressure, together with the limited dispersal ability of the aphid might be to a certain extent driving the dynamics of a metapopulation, in which not always all suitable patches are colonized, and colonization of suboptimal habitat can occur [34]. *Acyrthosiphon svalbardicum *overwinters as an egg, which has been demonstrated to survive down to -30°C [35]. The lowest temperature recorded in the soil (recorded under 30 cm of snow in a south facing site), is greater than the recorded lower lethal temperature for the aphid in its overwintering stage. The effects of winter ground ice cover on egg hatching success has never been measured, although it has been demonstrated to reduce soil microarthropod community populations by up to 50%, and it has been suggested to have significant implications for the usually patchy distribution of these animals [5]. Between November 2008 to April 2009 Svalbard airport weather station recorded 49.77 mm (as snow) of precipitation and 8 winter warm spells (when air temperature rises above 0°C, which combined with subsequent sub-zero temperatures leads to the creation of winter surface-ice layers in permafrost areas) comprising a total of 22 days when the winter air temperatures rose above 0°C. In the same period the following year, 83.53 mm of precipitation were registered (half of them as rain), and 15 warm spells comprising 42 days in total [36]. This could have had an effect on eggs survival during 2009/2010 winter. Moreover it should be recognised that the snow pack is not a homogeneous column. Ice layers within the snow profile created by winter melt events, often including rain, are frequently present. Hence the passage of melt water through the snow pack is not uniform [37] and a combination of snow thickness and route of percolation of liquid phase water through the snow pack will influence the development of potential surface ice layers. Such surface ice layers are known to reduce the overwintering survival of oribatid mites and Collembola [5] and may have an effect on the hatching success of overwintering eggs. Nonetheless, these eggs appear to be very cold hardy [35] although duration of exposure to low temperature or the effect of icing was not assessed. Hence it is important to be aware that snow depth may have many additional effects that are correlated. Such variations in the characteristics of the snow pack may explain why snow depth in itself fails to predict accurately the distribution of the aphid.

Freeze-thaw cycles in early spring may challenge the soil invertebrate fauna, especially in alpine or sub-arctic regions [38]. While it is known that surface freezing events can occur throughout the year [39], freeze-thaw cycles following snow clearance are less likely to occur in the high Arctic. Date of snow clearance is typically mid-June. At this date there has been midnight sun for over six weeks reducing soil diurnal temperature variation. As an example, in Adventfjord close to Longyearbyen the mean air temperature in June 2011 was 4.8°C [40] but the soil temperature at 5 mm depth only first became positive from 7^th ^June. By June 11^th^, soil temperature had attained 5°C with maximum temperatures over 13°C occurring during the month. No after melting soil freeze-thaw events were observed (Coulson SJ: Soil temperature records from Adventdalen, unpublished). Whilst we cannot exclude the possible effect of freeze-thaw cycles on egg and fundatrix survival it would appear that such events are probably not a major factor.

**Figure 7:**
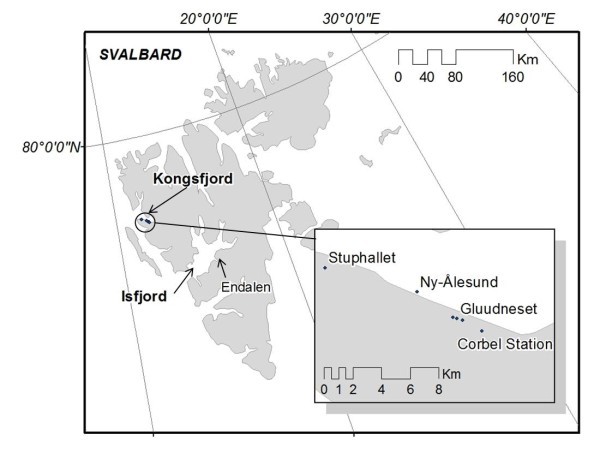
**Study location in Kongsfjord (Svalbard archipelago)**.

Snow depth has been shown to have a strong inter-annual variability due to factors such as wind direction, precipitation and temperature. 2010 was an unusual winter with heavy snow falls followed by strong repeated thawing events which resulted in a lesser overall snow accumulation and a thick ice layer underneath the snowpack. Even though 2010 was an unusual winter, these extremes are likely to be periodically experienced by the aphid population. In the case of a species with low dispersal ability such extreme events are likely to strongly influence distribution limits. Dispersal abilities of the aphid however could have also been underestimated. The species was previously hypothesized to be unable to rapidly track suitable environmental conditions and to be restrained to those few consistently microclimatically favourable sites [23]. However, the aphid was scored in 2010 in patches that were empty in 2009 and vice versa, which could indicate colonization and extinction events. Moreover, the aphid range has expanded since 1995. Whereas presence of the aphid was confirmed in the inner part of the fjord at the Corbel Station in 1995, the newly recorded presence of the aphid on coastal ridge on Stuphallet (Figure 7) accounts for an increase in the distribution range by up to 4.7 km from the distribution described in Kongsfjord, when the outermost location where the aphid was found was Brandalpynten, adjacent to Ny-Ålesund (Figure 7). Therefore, the possibility of local movement of aphids between suitable patches within a growing season is not to be underestimated. This observation agrees with previous studies on host-specific insect herbivores which indicated that species that show a restricted distribution within one host plant species may respond to environmental changes faster than the host plant itself [41]. Furthermore, alate occurrence in *A. svalbardicum *could strongly influence the dispersal abilities of the species, and potentially modify the distribution of the species over a short time. The aphid has been demonstrated to respond rapidly, within days, to increased summer temperatures [42] showing up to a 20-fold increase in population density in response to warming [43]. This positive effect on population density may, however, have an upper limit as the optimum temperature is exceeded, or increasing the density of predators and parasitoids [22]. Lack of density data within this survey however prevents analyzing the data in terms of population size and aphid density per patch. Hullé *et al*. [12] pointed out that during the period between 1992-2006 the summer thermal budget for the species has increased by 180 degree days, sufficient to produce the increase in egg production predicted by Strathdee *et al*. [6]. Field validation nonetheless, did not confirm this hypothesis as the extra generation was not found in a series of expected years [12]. Summer thermal budgets (degree days) for the aphid using the temperature logger data were not calculated since the logger sensor probes were not shielded from incoming solar radiation once the snow had cleared. Once the sensors were exposed to short wave radiation it is the response of the individual sensor to the incoming short wave radiation that is recorded. Therefore the temperatures recorded by the logger after snow melt are not representative of the microhabitat temperatures experienced by the aphid. However, an increase in the summer thermal budget, or the increase of temperature on the appropriate time of the season for the aphid development, could to an extent explain why the species was found in more patches under deep snow profiles than expected, but once again this would not explain the absence of the species in suitable patches.

## Conclusions

Snow depth can be used as a proxy for timing of snow melt in Kongsfjord for south facing slopes (Figure 1A and 1B). Moreover, a significant relationship between the presence of *A. svalbardicum *and winter snow depth was found, and consequently snow depth can be used as a predictor of the local distribution of the aphid. This is however just a coarse approximation. Strong inter-annual variation in snow depth (Figure 2) and correlation of snow depth with other variables such as plant phenology prevents finely predicting which patches will be occupied by the aphid. The relationships described here cannot define species distribution at landscape scale (Figure 3, 4, and additional file 2), given that they do not explain un-occupancy of patches under shallow snow depths with advance phenology (Figure 5 and 6). The dispersal ability of the species might have been underestimated in the past, since the distribution range of the species has shown an expansion of 4.7 km towards the fjord mouth from 1995. Variables able to accurately predict species distribution are difficult to define yet are important to understand, especially if projections of responses to climate change are to be modelled.

## Methods

### Study area

The coastal ridges of Gluudneset and Gåsebu are located in an approximately east-west orientation on the south shore of Kongsfjord, Spitsbergen (78.91°N 12.05°E) (Figure 7), a location where the aphid presence has been previously recorded [6]. Their main ridge areas face southwards, with a shorter and steeper north facing slope and only small east and west exposures. The top of the ridges show a typical polar semi- desert community dominated by *D. octopetala *whereas vegetation communities on the lower sections of the ridges are composed of a typical heath community dominated by *Cassiope tetragona *[18]. Mean daily air temperatures greater than 0°C are recorded from June to August and the average annual precipitation is around 371 mm yr^-1 ^mostly in form of snow in winter [42]. Soil summer temperatures have been showed to fluctuate between 0.6-16.1°C [44]. *Dryas octopetala*, the host plant of *A. svalbardicum*, is a circumpolar evergreen mat forming dwarf shrub species typical from, although not restricted to, dry calcareous soils which clear up of snow early in the season [19,45]. *Dryas octopetala *often does not grow higher than few centimetres over the ground, and usually occurs as individuals that can vary in size from a few centimetres to over a metre in diameter.

**Figure 8:**
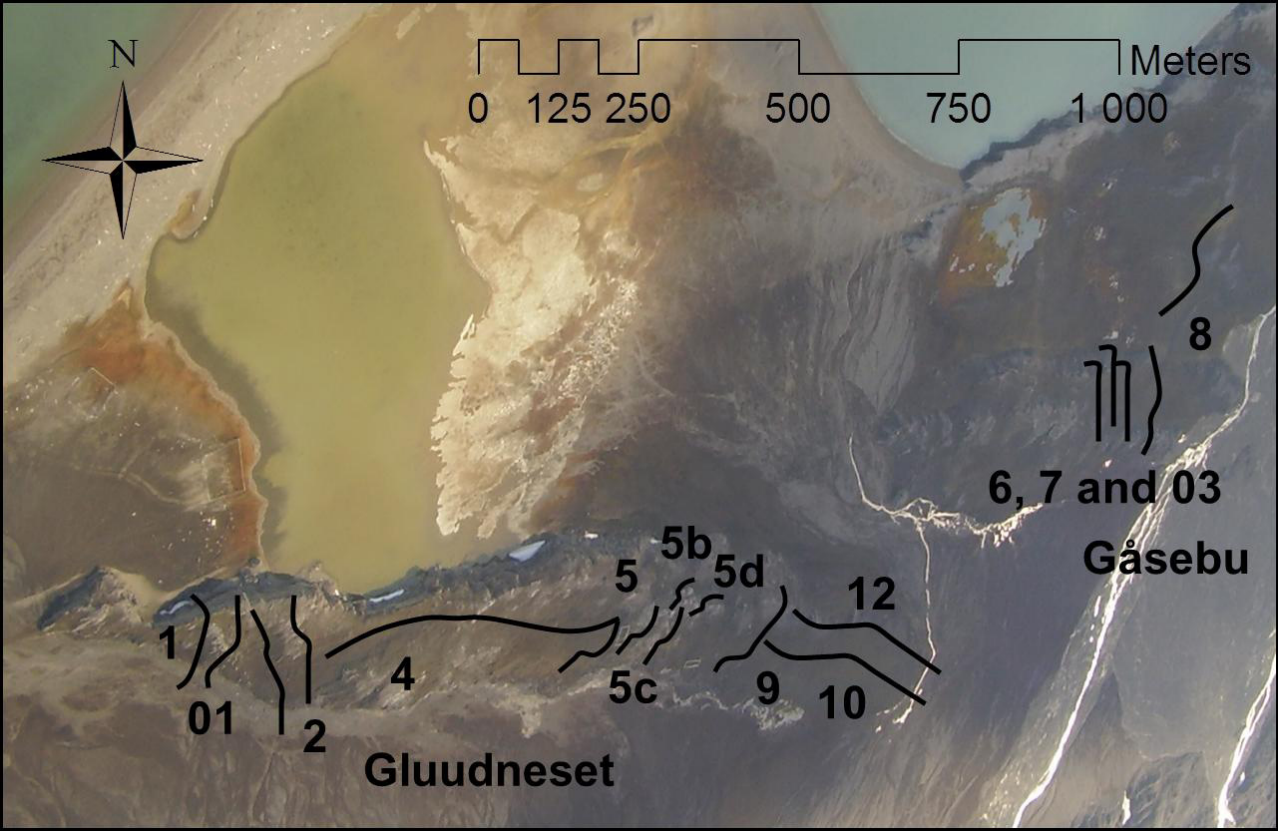
**Aerial photograph of the coastal ridges in Kongsfjord**. Superimposed transects show the location at Gåsebu and Gluudneset.

Snow depth and ice thickness (measured in early April), aphid site occupancy, and aphid and plant phenology (measured in August) were determined at the Gluudneset and Gåsebu coastal ridges. Measurements were taken in 2009 and 2010 along a series of transects (12 transects on Gluudneset ridge, and 4 transects on the Gåsebu ridge) (Figure 8) to cover the full range of snow depths in the area, from the shallowest to the deepest snow profile.

### Snow depth and ice thickness

Snow depth was measured using a 2.65 m long avalanche probe and was determined between 2^nd ^and 6^th ^April 2009 along a total of 16 transects (transects 1, 01, 2, 4, 5 and 5 a, b, c and d, 9, 10, and 12 on Gluudneset ridge, and transects 03, 6, 7, 8 on the Gåsebu ridge) (Figure 8), giving 594 data points with an approximate distance of 2 m between measurement points. Surface ice thickness was also measured (along each ridge by digging through the snow and the underlying ice layer if present until reaching the soil surface. The ice layer was measured with a ruler from the soil surface). The position of measurement locations were determined using a Differential GPS (DGPS) (Leica Geosystems SR20) and DGPS-position adjusted using the differential mode of the Leica Geosystems software. The position measured by the rover was adjusted to an accuracy of +/- 1 cm. In March 2010, two transects (6 and 5, one at each ridge) were re-visited and snow depth measured at each point.

### Timing of snow melt

Temperature fluctuations experienced at the soil surface are less severe under the snow pack than after snow melt due to the temperature buffering effect of the snow pack [33]. Such a buffering effect disappears as the snow melts and the sensors begin to measure a combination of air temperature and individual response to direct incoming short wave radiation. Date of snow clearance can be defined accordingly as the date the temperature data show an increase in the amplitude of daily fluctuations (See additional file 1). Five Tinytag temperature loggers (Gemini Data Loggers (UK) Ltd.) were placed on the ground surface under different snow depths (0, 10, 30, 100 and 130 cm) in south facing locations in April 2009 and collected the following August as proxy measurement of snow melt date. In March 2010, 10 additional Tinytag temperature loggers were placed on the ground surface in five south facing and five north facing locations under 10, 20, 30, 40 and 50 cm of snow, and recovered in July the same year. In all cases the loggers were placed on the soil surface under the snowpack and recorded temperature at intervals of 15 minutes.

### Aphid Phenology

Aphid phenology was assessed using a binocular microscope according to the system by Strathdee *et al*. [20], fundatrix, apterous viviparae, oviparae, male and egg. When present, the shiny black eggs are clearly visible on the underside of the leave and therefore it is not considered likely that they were overlooked.

### Host plant phenology

Plant phenology was recorded in a total of 282 patches in accordance with Strathdee *at al*. [46]: 1 (Stamens visible), 2 (Petals senescing), 3 (Seed head visible above petals), 4 (Seed head untwisting), 5 (Seed head twisting). If no bud was visible it was scored as 0. Sampling in August, although late in the season in relation to the aphid life cycle, was chosen so as to maximize the chances of detecting a relationship between delayed host plant phenology, aphid phenology and patch occupancy.

### Patch occupancy

Patch occupancy by *A. svalbardicum *(which patches of *D. octopetala *were colonized by the aphid) was recorded in early August at those points where *D. octopetala *was found at the exact same DGPS locations where snow depth was measured the previous spring.

**Figure 9:**
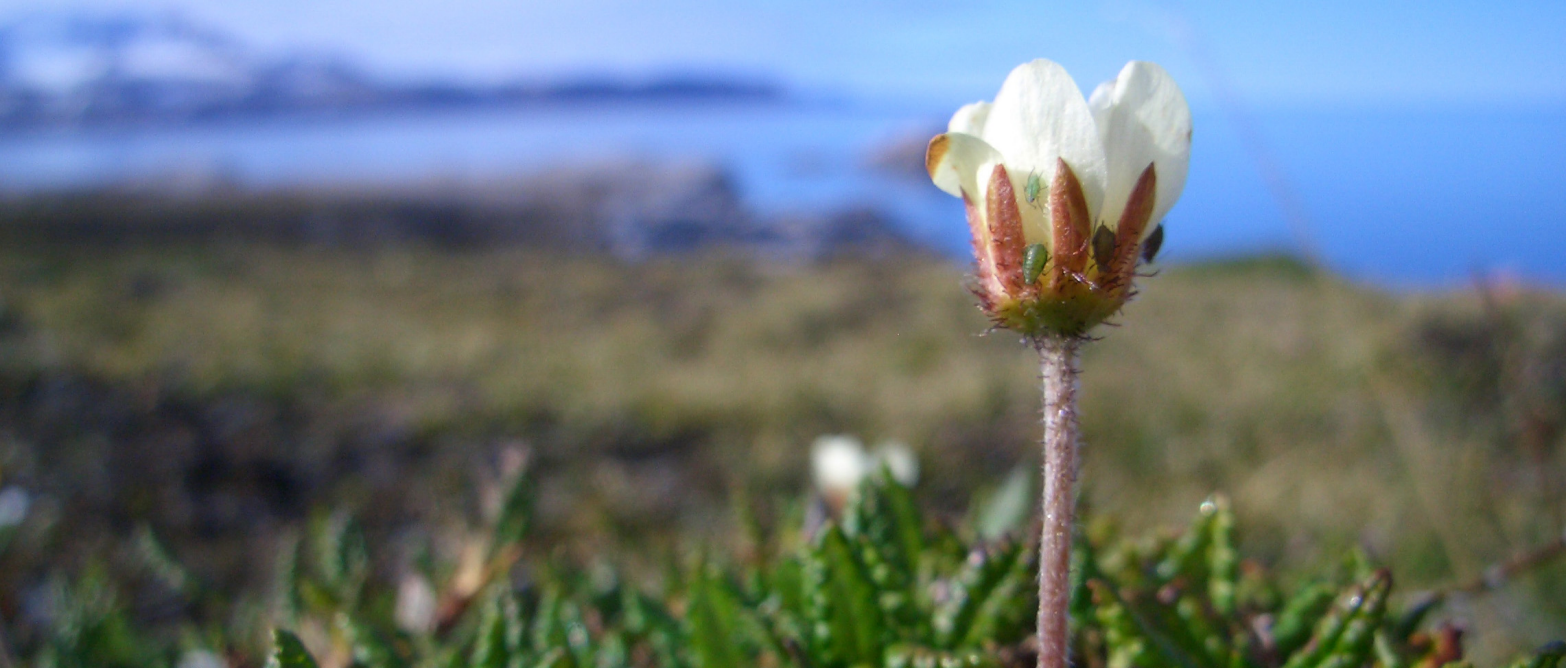
**Example of *D. octopetala *flower bud occupied by the aphid *A. svalbardicum***.

All flower buds and seed heads present in an area of 0.3 m radius from the DGPS position recorded in April were examined for aphid presence. In addition a sample of *D. octopetala *was taken at each patch by clipping approximately 2 cm of plant stem containing seed or, when present, flower buds (Figure 9). This sample was preserved in alcohol and examined under a binocular microscope at UNIS for the presence or absence of aphids and aphid phenology. Transect 5 was re-sampled in summer 2010 and, additionally, the end points surveyed by Strathdee & Bale [6] for aphid distribution were re-visited to assess for range expansion or contraction.

### Statistical analysis

Binomial linear model was carried out using R version 2.10.1 [47] with aphid presence/absence data as dependant variable and snow depth and plant phenology as environmental variables.

Mann-Whitney Rank-sum, Kruskall-Wallis tests and linear regression were performed using the statistical package included in SigmaPlot 11.0 (Systat Software Inc). Mann-Whitney Rank-sum test for non-parametric data was applied to test for relationships between snow depth and host plant phenology and between snow depth, plant phenology, and aphid life stage. Kruskall-Wallis one way analysis of variance on ranks for non- parametric data was applied 1) on the differences of snow depths covering different plant phenological stages; 2) to test for differences in the distribution of host plant phenological stages in relation to the orientation (south, north or top of the ridge); and 3) to the snow depth data grouped by plant phenological categories for occupied and non-occupied patches, with a *post-hoc *pairwise differences test performed implementing Dunn's method. Relationship between snow depth and timing of snow melt were tested using linear regression.

## Authors' contributions

SJC conceived the idea and contributed to the manuscript. Both MLAJ and SJC prepared the project manuscript funded by ARCFAC V and participated in the data collection. MLAJ led the data analysis and prepared the initial manuscript. All authors read and approved the final manuscript.

## Supplementary Material

Additional file 1 **Soil temperature recorded during 2010**. For each graph it is shown the soil temperature recorded during 2010 at different locations (north or south facing slope), and the winter snow depth under which the logger was placed. All loggers where placed on the soil surface under the snow and ice layer. The red arrow indicates the date of snow melt. Click here for file

Additional file 2 **Scaled distribution map along all measured transects on the Gluudneset and Gåsebu ridges with snow depth and host plant phenology**. Map shows the snow depths measured in April 2009 (blue circles), plant phenology recorded in August 2009 (0: No bud visible; 1: Stamens visible; 2: Petals senescing; 3: Seed head visible above petals; 4: Seed head untwisting; 5: Seed head twisting), and site occupancy recorded in August 2009 (Red triangles: occupied patches; Crosses: empty patches) in transects 9, 10 and 12. Solid lines delimit transects sketched in a modified position to fit the figure. Click here for file
