# Supplementary figures and images for: Can snow depth be used to predict the distribution of the high Arctic aphid Acyrthosiphon svalbardicum (Hemiptera: Aphididae) on Spitsbergen?
Source: BMC Ecol. 2011 Oct 13;11:25. doi: 10.1186/1472-6785-11-25 (PMC3208578; doi:10.1186/1472-6785-11-25)

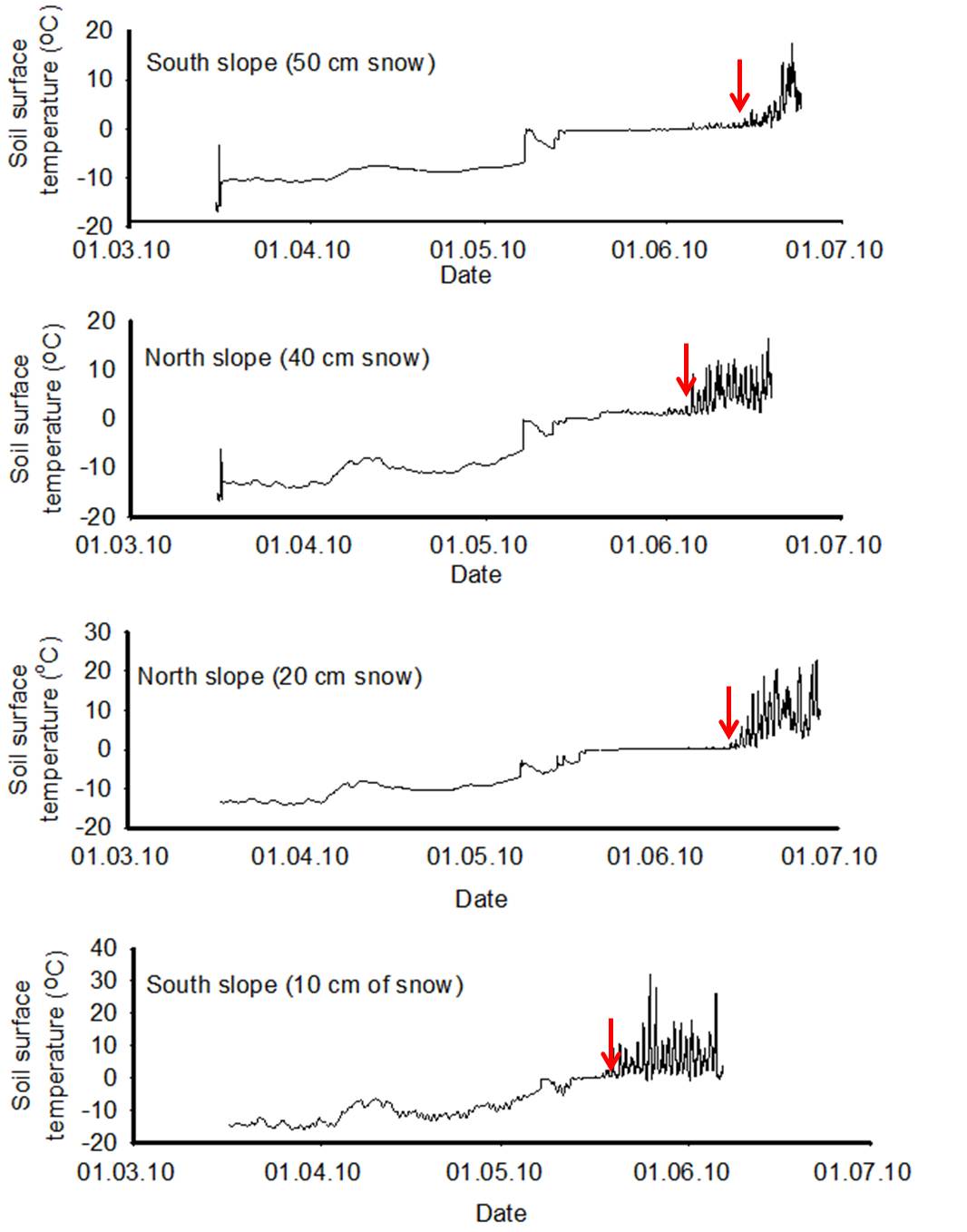

Supplement: Additional file 1 — Soil temperature recorded during 2010. For each graph it is shown the soil temperature recorded during 2010 at different locations (north or south facing slope), and the winter snow depth under which the logger was placed. All loggers where placed on the soil surface under the snow and ice layer. The red arrow indicates the date of snow melt. [file 1472-6785-11-25-S1.DOC]

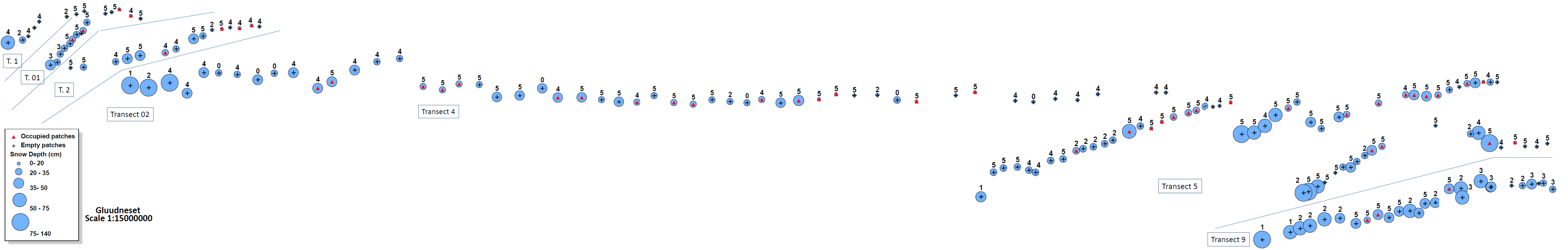

Transect 10

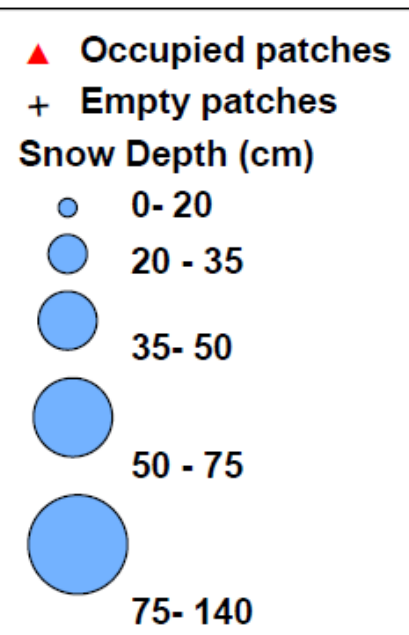

Transect 12

Gluudneset  
Scale 1:15000000

Transect 6, 7 and 03

Transect 8

Gåsebu

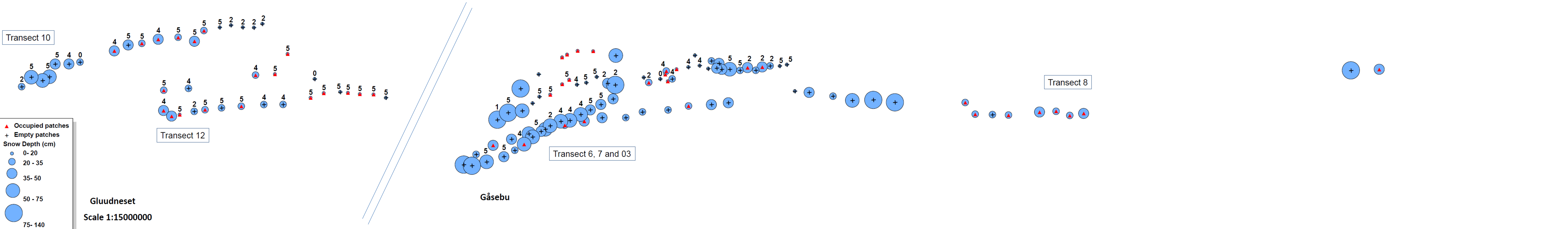

Supplement: Additional file 2 — Scaled distribution map along all measured transects on the Gluudneset and Gåsebu ridges with snow depth and host plant phenology. Map shows the snow depths measured in April 2009 (blue circles), plant phenology recorded in August 2009 (0: No bud visible; 1: Stamens visible; 2: Petals senescing; 3: Seed head visible above petals; 4: Seed head untwisting; 5: Seed head twisting), and site occupancy recorded in August 2009 (Red triangles: occupied patches; Crosses: empty patches) in transects 9, 10 and 12. Solid lines delimit transects sketched in a modified position to fit the figure. [file 1472-6785-11-25-S2.PDF]
